# Microplate Agglutination Test for Canine Brucellosis Using Recombinant Antigen-Coated Beads

**DOI:** 10.1155/2014/348529

**Published:** 2014-10-28

**Authors:** Yussaira Castillo, Masato Tachibana, Yui Kimura, Suk Kim, Yasuaki Ichikawa, Yasuyuki Endo, Kenta Watanabe, Takashi Shimizu, Masahisa Watarai

**Affiliations:** ^1^The United Graduate School of Veterinary Science, Yamaguchi University, 1677-1 Yoshida, Yamaguchi 753-8515, Japan; ^2^Laboratory of Veterinary Public Health, Joint Faculty of Veterinary Medicine, 1677-1 Yoshida, Yamaguchi 753-8515, Japan; ^3^Institute of Animal Medicine, College of Veterinary Medicine, Gyeongsang National University, Jinju 660-701, Republic of Korea; ^4^Merial Japan Limited, 3-20-2 Nishi Shinjuku, Shinjuku, Tokyo 163-1488, Japan; ^5^Laboratory of Small Animal Internal Medicine, Joint Faculty of Veterinary Medicine, Kagoshima University, 1-21-24 Korimoto, Kagoshima 890-0065, Japan

## Abstract

*Brucella canis,* a facultative intracellular pathogen, is the causative agent of canine brucellosis. The diagnosis of canine brucellosis is based on bacteriological examination and serological methods, including agglutination and gel diffusion tests. In this study, four recombinant antigens, heat shock protein 60, rhizopine-binding protein, Cu-Zn superoxide dismutase, and hypothetical protein (Ag 4), were constructed. These antigens were coated on latex beads and their usefulness in the serological diagnosis of canine brucellosis was examined. All recombinant antigens showed specific reaction with sera from *B. canis*-infected dogs in Western blotting. In a microplate agglutination test, mixing sera from *B. canis*-infected dogs, but not sera from *B. canis*-free dogs, with single or multiple antigens-coated latex beads produced clear agglutination. Moreover, the antigen-coated latex beads did not show nonspecific agglutination in hemolyzed serum samples. A survey of canine serum samples conducted by the microplate agglutination test using single antigen-coated latex beads showed that this method would be useful in the serological diagnosis of canine brucellosis. Further investigations using more serum samples are required to confirm the usefulness of our method.

## 1. Introduction

Brucellosis is one of the world's major bacterial zoonosis and an important cause of a serious debilitating disease in humans; further, it is the cause of abortion and sterility in animals.* Brucella canis*, a facultative, gram-negative, intracellular pathogen, is the etiologic agent of canine brucellosis [1]. Canine brucellosis is widely distributed around the world and this is an important disease due to the economic losses in animal production and its risks for human health [1]. Diagnosis of brucellosis is primarily based on bacteriological and serological tests [2]. Serological diagnosis is usually performed using a tube agglutination test (TAT), a rapid slide agglutination test, and a gel immunodiffusion test [2–4]. However, agglutination tests often give false positive results because of cross-reactions with other pathogens [2]. A general strategy for eliminating cross-reactions is to use purified antigen with unique epitopes in the serological tests. Bacterial cell wall antigens of* B. canis* can be prepared by hot saline extraction and are reportedly useful in serological diagnosis [5]. In a previous study, we reported the characterization of crude hot saline extracts and identified three antigens, namely, rhizopine-binding protein (RBP, former assigned ribose ABC transporter), Cu-Zn superoxide dismutase (SOD), and a hypothetical protein (Ag 4) [6].

This paper describes the development of latex beads coated with purified recombinant versions of these antigens and demonstrates the usefulness of these antigen-coated latex beads in the serological diagnosis of canine brucellosis.

## 2. Materials and Methods

### 2.1. Antigen Preparation

The gene encoding each antigen was amplified from chromosomal DNA isolated from* B. canis* [7] using PCR with the pair of primers shown in Table 1. The product was cloned into the pCold TF vector (Takara Bio Inc., Shiga, Japan). Trigger factor (TF) and His-tagged antigens were expressed in the* Escherichia coli* strain DH5*α*, and their purification was performed as described by the manufacturer (Novagen, Darmstadt, Germany) [8].

### 2.2. Canine Sera

Serum samples (*n* = 743) were collected from fleas- or ticks-infested dogs consecutively admitted to animal hospitals in Japan by the hospital staff in 2012 and 2013.* B. canis*-infected control sera were collected from three PCR-positive dogs, and* B. canis*-free control sera were collected from three specific pathogen-free (SPF) dogs [7].

### 2.3. Coating of Antigens onto Latex Beads

Covalent coupling of the proteins to carboxylated polystyrene latex beads was performed as described by the manufacturer (Polyscience Inc., Warrington, PA, USA). Briefly, equal volumes of antigen solution (protein concentration 300 *µ*g/mL, measured using Lowry method) and 2.5% latex beads (diameter 1 *μ*m, Polyscience Inc.) were mixed for 18 h at room temperature with stirring. Then, the latex beads were blocked with 0.2 M borate buffer containing 0.25 M ethanolamine for 30 min at room temperature with stirring and were washed three times with 0.2 M borate buffer containing 1% bovine serum albumin. The latex beads were then adjusted to a final concentration of 2.5% (w/v) with storage buffer and were maintained at 4°C.

### 2.4. Microplate Agglutination Test (MAT)

This test was performed by mixing equal volumes (50 *µ*L) of latex bead solution and canine serum diluted 50-fold with PBS in each well of a 96-well round-bottom microwell plate (Nunc, Roskilde, Denmark) and incubating the plate at room temperature for 18 h without shaking. Samples showing agglutination were considered positive.

### 2.5. TAT

This test was performed by adding equal volumes (0.5 mL) of heat-inactivated* B. canis* QE-13 whole-cell antigens (optical density of 0.8 at 450 nm, Kitasato Laboratories, Saitama, Japan), with serum that had been serially diluted 2-fold with PBS, and incubating the mixture at 50°C for 24 h. Agglutination titers were determined from the final dilution of serum showing 50% agglutination. Titer of samples showing higher than 160 was considered positive [9].

### 2.6. SDS-PAGE and Western Blotting

The antigen solution was separated using 10% SDS-PAGE and then transferred to Immobilon-P membranes (Millipore, Billerica, MA, USA). The efficiency of transfer was determined using Coomassie brilliant blue R-250, and then the membranes were tested for reactivity with antibodies in the* B. canis*-infected control sera that were collected from three PCR-positive dogs [6, 8]. Immunoreactions were visualized using the enhanced chemiluminescence detection system (GE Healthcare Life Science, Little Chalfont, UK).

## 3. Results

### 3.1. Reactivity of Recombinant Antigens with Canine Sera

**Figure 1:**
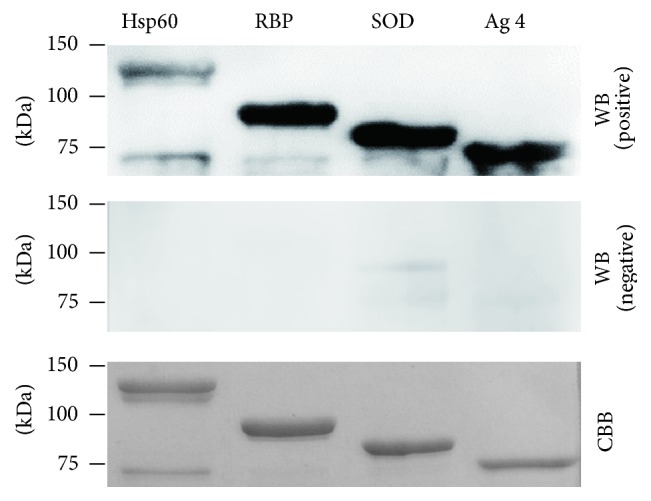
Western blot analysis of recombinant antigens. TF fusions with Hsp60, RBP, SOD, and Ag 4 were separated by SDS-PAGE under reducing condition and then transferred to nylon membranes. The membranes were stained with Coomassie brilliant blue (CBB) and were used for analysis of* B. canis*-infected (WB, positive) and *B.canis*-uninfected sera (WB, negative).

To test antigenic reactivity, four recombinant antigens were subjected to Western blotting along with the dog sera that had tested positive or negative in the TAT. All recombinant antigens showed strong reactivity with the positive sera, but not with the negative sera (Figure 1).

### 3.2. TAT

To identify* B. canis*-infected sera, we performed TAT on canine serum samples (*n* = 743) collected from dogs consecutively admitted to animal hospitals in Japan by the hospital staff. Antibodies to* B. canis* were detected in 9 of the 743 (1.2%) serum samples (Table 2). These TAT-positive sera did not show positive reaction in the PCR test.

### 3.3. MAT

**Figure 2:**
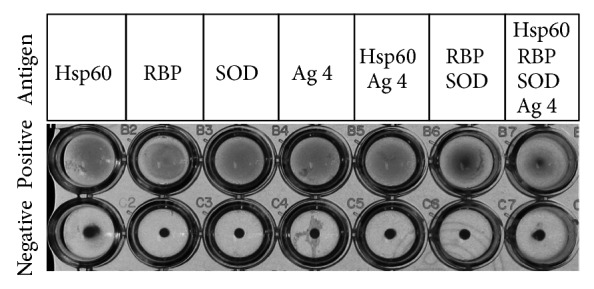
MAT using single or multiple antigens-coated latex beads. Antigens-coated latex beads were mixed with* B. canis*-infected (positive) and* B.canis*-uninfected (negative) sera.

Latex beads were coated with single or multiple recombinant* B. canis* antigens with the aim of achieving a serodiagnostic method that is faster and easier to perform. Sera from* B. canis*-infected dogs, in which infection had been confirmed by TAT and PCR previously, were mixed with the antigen-coated latex beads, and then this mixture was incubated at room temperature for 18 h. Agglutination was clearly observed in all antigens (Figure 2). In contrast,* B. canis*-free dog serum showed no agglutination (Figure 2). Various results were obtained when MAT was conducted using the 743 dog serum samples with each of the antigen-coated latex beads, as shown in Table 2. SOD reacted with 8 TAT-positive sera (88.9%), but RBP, Ag 4, and Hsp60 (heat shock protein 60) reacted with 6 (66.7%), 6 (66.7%), and 7 (77.8%) sera, respectively (Table 2). These antigens showed different reactivity in 4 of the 9 TAT-positive sera.

### 3.4. Elimination of False Positives by MAT

**Figure 3:**
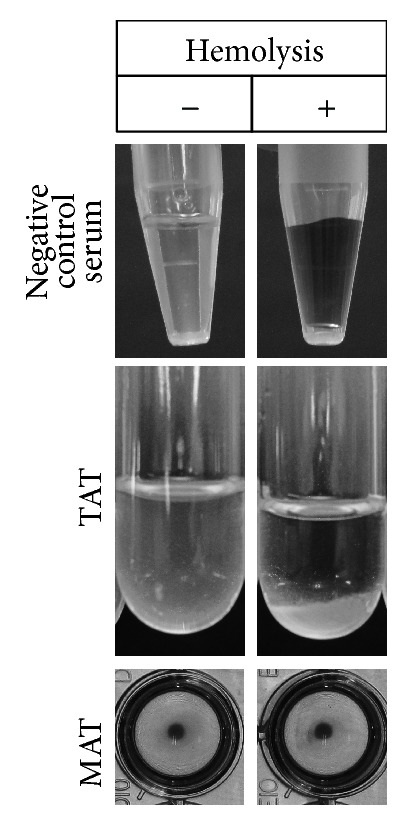
Elimination of false positive results using the MAT.* B. canis*-uninfected control sera were hemolyzed by freeze-thaw (upper panels). TAT (middle panels) and MAT were performed using recombinant RBP-coated latex beads (lower panels) in hemolyzed (+) or nonhemolyzed (−) sera.

It is well known that hemolysis of serum samples induces false positive results in TAT [10]. To investigate if this source of false positive results is eliminated in the MAT, we performed the assay with antigen-coated latex beads using three artificial hemolytic sera from* B. canis*-free dogs. Indeed, hemolyzed serum showed a false positive result in TAT (Figure 3). In contrast, MAT with RBP-coated latex beads gave a negative result with the hemolyzed serum (Figure 3). The same results were obtained with latex beads coated with other antigens (data not shown).

## 4. Discussion

TAT and MAT, using whole* B. canis* antigens, are currently being used for serological diagnosis in dogs in Japan [11, 12]. However, nonspecific reactions occur in the serological diagnoses using whole bacterial cell antigens [13]. In a previous study, we reported a rapid slide agglutination test using latex beads that were coated with antigens extracted by hot saline for the serological diagnosis of canine brucellosis [6]. To develop a serological diagnosis method that is faster and easier to perform, we applied latex beads coated with crude antigens. However, we observed nonspecific reaction in MAT and, therefore, we used latex beads coated with purified recombinant antigens in this study. Analysis of the antigens contained in the crude hot saline extracts, performed in our previous study, identified three antigens, namely, RBP, SOD, and Ag 4 [6]. Rhizopines are known to be nutritional mediators and play a role in nitrogen-fixing symbiotic associations between members of the Rhizobiaceae and legume plants [14]. Because* B. canis* is not a plant symbiont, the function of RBP of* B. canis* is completely unknown. In addition, the function of Ag 4 is currently unknown, and because SOD is a known antigenic protein of* B. abortus* [15], its potential value as a vaccine for brucellosis prevention and as a diagnostic reagent for the disease has been investigated [9, 16, 17]. The results from our previous study also showed that recombinant SOD was useful for the detection of canine brucellosis using ELISA [8]. Hsp60 has also been known as an immunoreactive protein and virulence factor of* B. abortus* [18]. The present study showed that these four recombinant antigens can be used as a potential diagnostic marker for canine brucellosis. In particular, because the SOD antigen showed the highest reactivity to TAT-positive sera in the MAT, recombinant SOD may be most useful for serological diagnosis. Usually, serum samples taken from dogs living in Japanese household do not show a positive result for PCR but sometimes show a positive result for TAT. We think bacteremia of* Brucella* may be rare case in dogs that are kept at home in Japan [6–8].

Hemolysis of serum samples affects serological diagnosis and induces false positive results. Although nonspecific agglutination shows a relationship with hemoglobin concentration [10], its detailed mechanism is still unclear. The purified recombinant antigen-coated latex beads developed in the present study will be useful tools for the serological diagnosis of canine brucellosis from hemolyzed serum specimens. Furthermore, MAT can be used for very small serum samples and offers potential for high throughput testing; therefore, it would be suitable for the screening of valuable samples such as those from wildlife or small animals. Moreover, conventional serological and bacteriological tests would be required to diagnose canine brucellosis.

## Figures and Tables

**Table 1:** Primers used in this study.

Antigen	GenBank accession number	Primers (5′-3′)	Reference
Hsp60	ABX63389.1	F: ATGGCTGCAAAAGACGTAAAA	This study
		R: TTAGAAGTCCATGCCGCCCAT	

RBP	ABX64027.1	F: ATGTTCAAGAAGGGTATGCGC	This study
		R: TTACTGTTCCAGAACGAACGG	

SOD	ABX63876.1	F: ATGAAGTCCTTATTTATTGCA	[16]
		R: TTATTCGATCACGCCGCAGGC	

Ag 4	ABX61923.1	F: ATGACCACTGGCATGGATGAC	This study
		R: TCAATCCTGCTTCACCTGACG	

**Table 2:** Serological analysis of canine sera.

Antigen	TAT+		TAT−	
	9/743 (1.2%)		734/743 (98.8%)	
	MAT+	MAT−	MAT+	MAT−
Hsp60	7 (77.8%^a^)	2 (22.2%)	0 (0%)	734 (100%^b^)
RBP	6 (66.7%^a^)	3 (33.3%)	0 (0%)	734 (100%^b^)
SOD	8 (88.9%^a^)	1 (11.1%)	0 (0%)	734 (100%^b^)
Ag 4	6 (66.7%^a^)	3 (33.3%)	0 (0%)	734 (100%^b^)

^a^Sensitivity; ^b^specificity.
